# Characterization of Translationally Controlled Tumour Protein from the Sea Anemone *Anemonia viridis* and Transcriptome Wide Identification of Cnidarian Homologues

**DOI:** 10.3390/genes9010030

**Published:** 2018-01-11

**Authors:** Aldo Nicosia, Carmelo Bennici, Girolama Biondo, Salvatore Costa, Marilena Di Natale, Tiziana Masullo, Calogera Monastero, Maria Antonietta Ragusa, Marcello Tagliavia, Angela Cuttitta

**Affiliations:** 1 National Research Council-Institute for Marine and Coastal Environment (IAMC-CNR), Laboratory of Molecular Ecology and Biotechnology, Detached Unit of Capo Granitola, Via del mare, 91021 Torretta Granitola (TP), Sicily, Italy; carmelo.bennici@iamc.cnr.it (C.B.); girolama.biondo@iamc.cnr.it (G.B.); marilena.dinatale@iamc.cnr.it (M.D.N.); tiziana.masullo@iamc.cnr.it (T.M.); linda.monastero@iamc.cnr.it (C.M.); marcello.tagliavia@iamc.cnr.it (M.T.); 2 Department of Biological, Chemical and Pharmaceutical Sciences and Technologies, University of Palermo, Viale delle Scienze, Ed. 16, 90128 Palermo, Sicily, Italy; salvatore.costa@unipa.it (S.C.); maria.ragusa@unipa.it (M.A.R.)

**Keywords:** cnidarians, transcriptome wide analysis, translationally controlled tumour protein, TCTPs, comparative genomics, homology modelling, gene expression

## Abstract

Gene family encoding translationally controlled tumour protein (TCTP) is defined as highly conserved among organisms; however, there is limited knowledge of non-bilateria. In this study, the first TCTP homologue from anthozoan was characterised in the Mediterranean Sea anemone, *Anemonia viridis*. The release of the genome sequence of *Acropora digitifera*, *Exaiptasia pallida*, *Nematostella vectensis* and *Hydra vulgaris* enabled a comprehensive study of the molecular evolution of TCTP family among cnidarians. A comparison among TCTP members from Cnidaria and Bilateria showed conserved intron exon organization, evolutionary conserved TCTP signatures and 3D protein structure. The pattern of mRNA expression profile was also defined in *A. viridis*. These analyses revealed a constitutive mRNA expression especially in tissues with active proliferation. Additionally, the transcriptional profile of *A. viridis* TCTP (*AvTCTP*) after challenges with different abiotic/biotic stresses showed induction by extreme temperatures, heavy metals exposure and immune stimulation. These results suggest the involvement of *AvTCTP* in the sea anemone defensome taking part in environmental stress and immune responses.

## 1. Introduction

The translationally controlled tumour protein (TCTP) represents a conserved protein that is widely expressed among eukaryotes. Members of TCTP family are 21–23 kDa proteins that do not share any similarity with other known protein families [1], while analyses of TCTP 3D structure from the yeast *Schizosaccharomyces pombe* revealed a fold similar to a small chaperone [2]. It consists of four β-sheets, named A–D and three principal helices, known as H1–H3, which are connected in a complex topology [2]. Sequence and structural comparison between members of TCTP protein family revealed that differences among structure mainly relies in a flexible loop located between the core and the helical domain [2].

TCTP homologues have been characterised in a variety of invertebrates, these include the nematodes *Brugia malayi* [3] and *Caenorhabditis elegans* [4], the crab *Eriocheir sinensis* [5], the shrimps *Litopenaeus vannamei*, *Penaeus indicus*, *Penaeus monodon* and *Marsupenaeus japonicas* [6,7,8,9]. Recently, members of the TCTP family have also been identified in marine bivalves such as *Venerupis philippinarum* [10], *Mytilus galloprovincialis* [11] and *Chlamys farreri* [12]. TCTP proteins are ubiquitous expressed to exert diverse physiological activities; indeed, they play a role in different cellular pathways such as cell growth, cell cycle progression and differentiation, gene regulation, stress/immune response, apoptosis and it has been also ascribed to TCTP an extracellular role cytokine-like [13,14]. However, there is little characterisation of TCTP genes in non-bilaterians as a single homologue has been described in *Hydra vulgaris* [15] and to date, even if identified, no homologues have been characterised in other cnidarians.

The sea anemone *Anemonia viridis* is a common Mediterranean cnidarian [16,17]. The phylum Cnidaria is a sister to Bilateria within the Eumetazoan, a phylogenetic split that dates back to the Precambrian era [18]. The phylum split into two major lineages: the class Anthozoa to which *A. viridis* belongs and its sister group, the Medusozoa. Combined analyses of morphological characters and molecular data are consistent with the idea that the Anthozoans are basal within the cnidarian phylum and they best represent the primitive cnidarians [19,20]. Despite the occurrence of a single body axis and a diploblastic organisation, genomic and transcriptional analyses revealed unexpected ancestral complexity [21,22,23]. Extant cnidarians represent the first organisms from organized cells into tissues [24] and as sister group of the Bilateria they are of great interest for the identification of mechanisms leading to bilaterian traits [25,26,27].

Several cnidarian transcriptome datasets have been generated during the years [21,22,28,29,30] and have permitted the use of bioprospecting and computational analysis [31,32,33]. In the present work, an open reading frame (ORF) for a TCTP family member, herein designated as *A. viridis* TCTP (*AvTCTP*), was recovered from *A. viridis* and its genomic organisation was characterised. An extensive transcriptional and genomic survey for the mining of expressed TCTPs from the stony corals (*Acropora millepora* and *Acropora digitifera*), the sea anemones (*Nematostella vectensis* and *Exaiptasia pallida* in addition to *A. viridis*) and *H. vulgaris* was carried out to analyse the molecular evolution of the TCTP gene family among cnidarians.

Based on advances in homology recognition and using modern modelling and comparative tools, the 3D structures of representative cnidarian TCTPs were predicted and compared in order to provide structural and evolutionary details. Moreover, the gene expression pattern of *AvTCTP* in different sea anemone districts and in response to abiotic and immune challenges was evaluated.

## 2. Materials and Methods 

### 2.1. Ethics Statement

*A. viridis* housing and husbandry were performed in accordance with the best practices developed in the cnidarian community to optimise animal health. All experiments were carried out in compliance with local laws and to date no specific permit is required for the performed experiments. However, all facilities and procedures complied with the Directive 2010/63/EU and the Animal Research: Reporting of In Vivo Experiments (ARRIVE) guidelines.

### 2.2. Data Mining

The characterized TCTPs were used initially to retrieve their corresponding sequences from the publicly available database at the National Centre for Biotechnology Information (NCBI). To obtain more homologs, these sequences were used as query to perform extensive protein-protein BLAST (BLASTP), nucleotide BLAST (BLASTX) and protein-nucleotide BLAST (TBLASTN) searches until no novel putative matches could be retrieved. The putative matching sequences from *A. millepora*, *A. digitifera*, *A. viridis*, *N. vectensis* and *H. vulgaris* were obtained from public available databases under the following accession numbers: XP_015766473.1 (*A. digitifera*), JR988252.1 (*A. millepora*), FK734027.1 and FK724838.1 (*A. viridis*), XM_021056176.1 (*E. pallida*), DV094372.1 and XM_001624752.1 (*N. vectensis*), NM_001309745.1 (*H. vulgaris 1*) and XM_002157314.3 (*H. vulgaris 2*). All the corresponding genomic sequences were retrieved similarly with the exception of *AvTCTP* which has been isolated experimentally. Because the existence of numerous gap in the *N. vectensis* genomic scaffold NEMVEscaffold_304, it was not possible to unambiguously assign gene structure. Thus, the version to date annotated has been used.

### 2.3. DNA, RNA Extraction and cDNA Synthesis

Specimens of *A. viridis* were manually collected from the Capo Granitola Coast (Torretta Granitola, Latitude 37°34′30.00″ N Longitude 12°40′47.26″ E) in the South of Sicily (Italy) and maintained in Millipore Filtered Sea Water (MFSW) at 18 ± 1 °C with a 12:12 h light:dark photoperiod. Sea anemones were acclimated for 20 days prior to challenges.

During acclimation, water parameters (pH, salinity, temperature, dissolved oxygen, turbidity, conductivity, ammonium, ammonia, chloride and nitrate levels) were continuously checked to measure the water quality of the aquarium through YSI ProDSS multiparameter probe (626870-1) (Dublin, Ireland). Moreover, health of the sea anemones was monitored through continuous observation.

For RNA purification, tentacles, pharynx, basal disk and body column and mesenterial filaments were rapidly recovered. Tissues were frozen in liquid nitrogen and ground into a fine powder using a tissue disruptor. The powder was dissolved in Trizol reagent (Invitrogen Corporation, Carlsbad, CA, USA) and further RNA purification steps were performed according to the manufacturer’s instructions. RNA concentrations and quality were verified by spectrophotometry (optical density (OD) at 260 nm), whereas the RNA integrity was checked using a 1.5% agarose gel. The RNA was stored at −80 °C for future use. For DNA extraction, the powder was dissolved in a DNAzol reagent (Invitrogen Corporation) and further genomic DNA purification steps were performed according to the manufacturer’s instructions. DNA concentrations and quality were verified by spectrophotometry (OD at 260 nm), whereas the integrity was checked using a 0.8% agarose gel. The DNA was stored at −20 °C for future use.

The extracted RNA (2 μg) was treated with RNA qualified 1 (RQ1) RNase-Free DNase (Promega, Madison, WI, USA) to remove any residual genomic DNA contamination and the DNase was inactivated by adding 25 mM EDTA. First-strand cDNA was synthesised from 2 μg DNase-treated total RNA samples using oligo(dT)_18_ and Superscript III (Invitrogen Corporation) following the manufacturer’s instructions. The cDNA mixture was stored at −20 °C. 

### 2.4. Full-Length cDNA and Gene Cloning from Anemonia viridis

Based on the partial sequence of *AvTCTP* cDNA, the 3′ end was obtained by PCR-RACE (rapid amplification of cDNA ends) using the SMART RACE cDNA application kit (Clontech, Mountain View, CA, USA) and the 3′ *AvTCTP* primer (Table 1) as described in the user manual. The 3′ RACE product was cloned into the pGEM-T Easy vector (Promega, USA) and transformed into XL1- Blue *Escherichia coli* cells (Stratagene, San Diego, CA, USA). Plasmid DNA, from three independent clones, was purified on Illustra™ plasmidPrep Mini SpinKit (GE Healthcare Life Sciences, Chicago, IL, USA) and sequenced using T7 and SP6 primers.

The full-length cDNA, consisting in sequences from original expressed sequence tags (ESTs) and additional elements from 3′ RACE product was amplified using an appropriate pair of primers (FlTCTP Fw/Rv) cloned in into the pGEM-T Easy vector (Promega) and the nucleotide sequence was verified using T7 and SP6 sequencing primers. Based on the 5′- and 3′-UTR (untranslated region) sequences, the primer set FlTCTP Fw/Rv was used to isolate the genomic DNA sequences.

### 2.5. Sequence and Phylogenetic and Structural Analyses

ConSurf webserver [34] was used to construct and map the evolutionary variability of nucleotides onto the secondary structures of the 5′-UTR from *AvTCTP*. Functional sites and domains in the predicted amino acid sequences were predicted using the Simple Modular Architecture Research Tool (SMART) program, the InterPro database [35], the Pfam database [36], the PROSITE program [37] and the Eukaryotic Linear Motif resource (ELM) for Functional Sites in Proteins. 

To reconstruct the molecular evolution of the TCTP family Basic Local Alignment Search Tool (TBLASTN, BLASTP) analyses were performed to recover TCTPs from GenBank (Table 2). TCTP protein sequences were aligned using T-Coffee software (http://tcoffee.crg.cat/apps/tcoffee/index.html) [38]. Phylogenetic and molecular evolutionary analyses were conducted using a Maximum Likelihood (ML) method, implemented in MEGA version 7.0 [39] in which Poisson correction, pairwise deletion and bootstrapping (1000 replicates) were considered as parameters, to reconstruct the evolutionary diversification and the molecular evolution of TCTP proteins family in cnidarians and different Eumetazoan groups. Moreover, the 3D structures of TCTP proteins were reconstructed exploiting the advances in homologues detection [23,27,40]. Thus homology modelling via the Protein Homology/analogY Recognition Engine 2.0 (Phyre 2) software [41] using the intensive modelling mode was carried out. Candidate structures for homology modelling were selected according to pairwise alignment. At least two different structures were used as a template for each generated structure and homology models were built for all the sets of proteins. Validation of the structural protein models was performed by assessing the Ramachandran plots. Cycles of clash minimization were also performed for the refinement of structures. Secondary structures assignments and relative solvent accessibility (RSA) were calculated by the DSSP program [42,43] as implemented in ENDscript [44]. Additionally, ConSurf webserver [34] was used to map the evolutionary variability of amino acids onto the reconstructed structures of homologues which were rendered using the UCSF Chimera package [45].

### 2.6. Anemonia viridis Translationally Controlled Tumour Protein Tissue-Specific Expression Pattern

Reverse Transcription-PCR (RT-PCR) was used to profile the tissue specific mRNA expression of *AvTCTP.* The cDNA from the different anatomical district previously synthetized were used as templates, whereas qTCTP-F and qTCTP-R (Table 1) primers were used. PCR amplifications were performed using Platinum Taq DNA Polymerase (Thermo Fisher Scientific Inc., Carlsbad, CA, USA) under the following conditions: pre-incubation at 95 °C for 2 min; 35 cycles consisting of denaturation at 95 °C for 30 s, annealing at 55 °C for 15 s and extension at 72 °C for 15 s; and a final extension at 72 °C for 1 min. The amplified products were analysed on a 1.7% agarose gel. The full length AvTCTP cDNA and gene amplicon were amplified under the following conditions: pre-incubation at 95 °C for 2 min; 35 cycles consisting of denaturation at 95 °C for 30 s, annealing at 55 °C for 30 s and extension at 72 °C for 1 min; and a final extension at 72 °C for 5 min.

### 2.7. Challenging Sea Anemone with Environmental Elicitors

To test the effect of hyperthermic conditions on *AvTCTP* expression, sea anemones (9 animals in the experimental group at 28 °C and 9 animals in the control group at 18 °C) were maintained in MFSW for variable lengths of time (from 6 h to 48 h) with a 12:12 h light:dark photoperiod. Two tentacles from 3 randomly selected animals were dissected after 6, 24 and 48 h from animals in experimental and control groups. Dissected tentacles were used for RNA purification procedures and quantitative RT-PCR (RT-qPCR) assays. After tentacle’s collection, specimens were removed and held in separate aquaria.

To test the effect of heavy metals on *AvTCTP* expression, sea anemones (3 animals per treatment; 9 animals per metal) were subjected to 12-h waterborne exposure to CdCl_2_ and PbCl_2_ at 18 ± 1 °C with a 12:12 h light:dark photoperiod. Animals (*n* = 3) maintained in MFSW were used as controls. The CdCl_2_ and PbCl_2_ solutions were prepared using 99% pure chloride salts (Sigma-Aldrich, St. Louis, MO, USA). Stock solution were prepared in Milli-Q water and diluted in MFSW to 2, 10 and 50 µg/L. Two tentacles were dissected from each animal at 12 h of exposure and used for RNA purification and RT-qPCR assays.

To explore the influence of immune challenges on *AvTCTP* expression, lipopolysaccharides (LPS) from *Pseudomonas aeruginosa* serotype 10 (L7018, Sigma-Aldrich) and peptidoglycans (PGN) from *Bacillus subtilis* (69554, Sigma-Aldrich) were dissolved in sterile Hank’s Balanced Salt Solution (HBSS, pH 7.4) at concentrations of 100 μg/mL. A volume corresponding to 200 μL of each immunogen was injected via a 27G needle in the basal disk of sea anemones. Sea anemones injected with 200 μL of HBSS were used as the control. Experimental and control groups (9 animals for LPS challenge, 9 animals for PGN challenge and 9 animals for control experiment) were maintained at 17 °C in MFSW at a 12:12 h light:dark photoperiod. Portions of the basal disk, including the injection site, were collected at 6 h, 24 h and 48 h and stored at −80 °C until used. After collection of basal disk, specimens were removed and held in separate aquaria.

### 2.8. Quantitative Reverse Transcription PCR (RT-qPCR)

RT-qPCR was performed using the ABIPRISM 7500 System (Applied Biosystems, Forster City, CA, USA) with Power Sybr Green as detection chemistry (Applied Biosystems).

RCC2 (Regulator of Chromosome Condensation protein 2) and COP-γ (Coatomer subunit gamma) were selected as control genes based on their expression stability in all tested conditions and the normalization factor was calculated using the GeNorm software [46]. Serial dilutions of pooled cDNAs from both control and treated samples were prepared to determine the PCR efficiency of the target and reference genes (data not shown) and amplification efficiency ranged from 1.9 to 2.2. All the primer sequences used in this study are listed in Table 2. RT-qPCR was conducted according to the manufacturer’s recommended procedures and every reaction was repeated in triplicate. The amplification conditions were the following: initial denaturation at 95 °C for 10 min and 40 cycles of 95 °C for 30 s and 60 °C for 50 s, followed by a melting curve from 60 to 95 °C. Amplicons were detected by agarose gel analysis after each PCR to confirm the amplification of the specific gene. All data represented relative mRNA expressed as the mean ± standard deviation (SD) (*n* = 3). Significant differences between values of different treated groups and the reference control groups were determined by one-way ANOVA with Tukey’s post-test.

## 3. Results and Discussion

### 3.1. Anemonia viridis cDNA Characterisation and Identification of TCTPs in Cnidarians

Starting from two partially overlapped sequences (FK724838.1 and FK734027.1) in the EST databases of the sea anemone *A. viridis*, a specific primer targeting the position between 612 and 632 nucleotides (nt) (numbering refers to the full-length cDNA) was designed and used to isolate the 3′-end of the cDNA. The putative full-length cDNA was obtained by assembling the 3′ RACE product with the original sequences and validated by sequencing. The full-length cDNA was 959 bp with a 5′-UTR of 290 bp and a 3′-UTR of 126 bp. 

**Figure 1:**
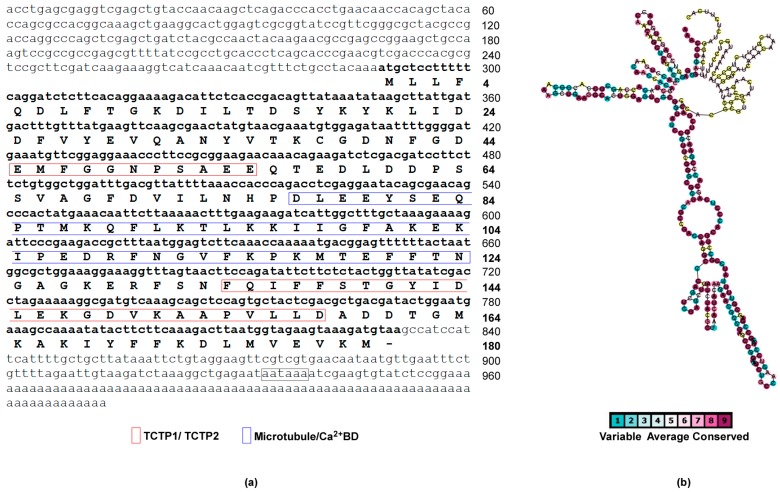
Full-length cDNA and protein sequences characterisation of sea anemone *Anemonia viridis* TCTP (AvTCTP). (**a**) The nucleotide and deduced amino acid sequence of the open reading frame (ORF), 5′- and 3′-untranslated regions (UTRs) were numbered on the right. The amino acids that constitute the TCTP signature and microtubule/Ca++ binding region are shown. (**b**) The secondary structure of 5′-UTR of *TCTP* mRNA from *A. viridis* was calculated to form high order of conserved secondary structures with extensive stem loops. Variable positions are presented in light blue; while conserved amino acids are shown in purple as defined in the color-coding bar.

Four in-frame stop codons (TAG) and a Kozak consensus (AAAATGC) are contained in the 5′-UTR upstream of the ATG start codon, while, a stop codon (TAA), a polyadenylation signal (AATAAA) and a poly(A) tail were found in the 3′-UTR (Figure 1a). Moreover, the 5′-UTR possesses high CG-content (nearly 60%) which is responsible for the generation of several configurations of secondary structures and with high degree of conservation in nucleotide position supporting selected RNA folds (Figure 1b). It has been demonstrated that extensive secondary structures enable the activation of double-stranded (ds) RNA-dependent protein kinase PKR allowing translational regulation of TCTP in mouse [47]. Thus, it is possible to hypothesize that analogous mechanisms could regulate TCTP expression in sea anemones establishing a regulatory system which has been conserved through the evolutionary time [18].

The cDNA contains an ORF of 543 bp corresponding to 180 amino acid residues. The predicted protein has an estimated molecular mass of 20,553.68 Da, with a theoretical isoelectric point (pI) of 4.54 and possesses the two TCTP signatures (TCTP1 located between residues 44–54; TCTP2 located between amino acid residues 131–158). The overlapping microtubule-binding region and the Ca^2+^ -binding motif were also identified.

A comprehensive exploration of the TCTP homologues in selected cnidarian species was performed led to the identification of 6 putative homologues were retrieved in these organisms (Table 3) in addition to *A. viridis* TCTP. Their identities were checked manually and the matching sequences were reconfirmed by comparative analysis.

Several other ORFs were detected in the datasets but they were not used for subsequent analysis as they contained in frame stop codons, no initiator ATGs, truncations, or they were considered likely to be an artefact because of the absence of any related ESTs. 

Two TCTP homologues (herein named as Hydra 1 and Hydra 2) were found in *H. vulgaris* whereas, our survey retrieved one matching sequence encoding putative TCTPs in both the sea anemones and stony corals. 

**Figure 2:**
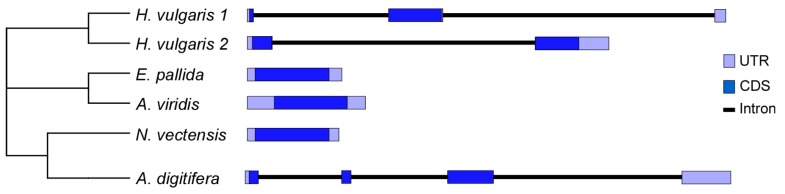
Gene structure for cnidarians TCTPs. The light boxes represent 5′- and 3′-UTR of the mRNA; while protein coding exons are shown in blue. Phylogeny of *H. vulgaris*, *E. pallida*, *A. viridis*, *N. vectensis* and *A. digitifera* based on data herein reported. CDS, coding DNA sequence.

The organization of *TCTP* genes were also reconstructed in order to analyse the molecular evolution of the TCTP gene family among cnidarians. A common gene structure was found in sea anemones because TCTPs from *A. viridis*, *N. vectensis* and *E. pallida* are encoded by intron-less genes. This gene organisation is different than that of the genomic structure of TCTPs in *A. digitifera* and *H. vulgaris* (Figure 2). 

In *A. digitifera* three introns interrupt the coding sequence with the first exon (121 bp) corresponding to the 5′ UTR, the translation initiation codon ATG and 9 amino acids of the N-terminus. The second (71 bp) and third (420 bp) exons include the TCTP signatures; whereas, the stop codon and 3′-UTR were found in the last exon (450 bp).

In Hydra 1, two introns interrupt the coding sequence with the first exon (54 bp) corresponding to the 5′-UTR, the translation initiation codon ATG and 10 amino acids of the N-terminus. The second (513 bp) exon includes the TCTP signatures, the stop codon and a few nt of 3′-UTR. The remaining sequences corresponding to the 3′-UTR mapped in the third exon (106 bp). Conversely, Hydra 2 gene structure includes the presence of two exons separated by a single intron. The first exon (234 bp) corresponds to the 5′-UTR, the translation initiation codon ATG and 56 amino acids of the N-terminus (including TCTP1 signature); whereas, the second exon (682) corresponds to the microtubule/Ca^2+^ binding domain, the amino acid residues of the C-terminus and the 3′-UTR.

The genomic structure of TCTPs orthologous from bilaterians is quite different. Comparative genomic analyses revealed that among Protostomes, molluscs as *Crassostrea virginica* and *Biomphalaria glabrata* possess five introns interrupting the coding sequence; while TCTP genes from nematodes as *Caenorhabditis elegans* or *Caenorhabditis remanei* possess two exons separated by a single intron. Similarly, gene structures consisting of four or five introns interrupting the coding sequences are usually found in Deuterostomia [48]. TCTP genes from tetrapods—including *Danio rerio*, *Xenopus tropicalis*, *Gallus gallus*, *Mus musculus* and *Homo sapiens*—showed comparable structures with the presence of six exons separated by five introns.

To date, two opposing theories on intron origin known as “intron early” and “intron late” hypothesis have been proposed. It has been suggested that the eukaryotic ancestors had intron-rich genes, with intron densities comparable to those in the most intron-rich modern genomes such as those of vertebrates [49]. During the first era of eukaryotic evolution, it has been suggested that evolutionary forces have acted to change the intron number leading to significant intron loss in most lineages [50]. Recently, it has been suggested that despite diploblastic organisation, Cnidarians are reduced Mesodermata, representing an important step in the early evolution of mesoderm and in tissue organisation [51], thus occupying a crucial position in animal evolution. Therefore, it is particularly striking that the repertoire of the cnidarians TCTPs displayed either intron-less genes or genes with spliceosomal introns. Moreover, it is possible to hypothesise that the dynamic of intron gain and loss may have occurred independently in each organism after the species diverged during the evolution.

### 3.2. Phylogenetic and Structural Analysis of Cnidarian TCTPs

**Figure 3:**
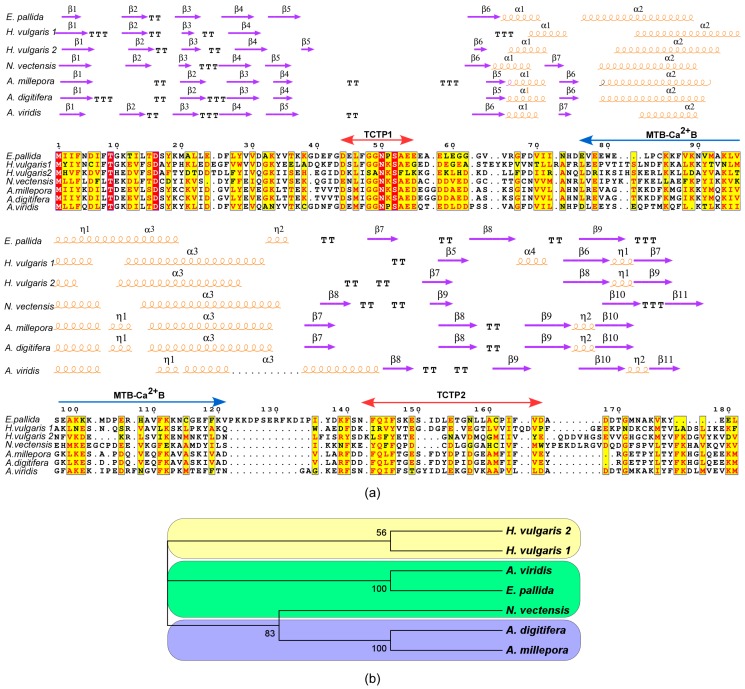
Phylogenetic relationship of Cnidarian TCTPs. (**a**) Multiple sequence alignment of the TCTPs in cnidarians. Alignment was performed with T-coffee. Similar residues are written in red bold characters and boxed in yellow whereas conserved residues are in white bold characters and boxed in red. The sequence numbering on the top refers to the alignment. (**b**) Phylogenetic tree based on the cnidarian TCTPs. The evolutionary history was inferred by using the Maximum Likelihood method based on the Poisson correction model. Internal branches were assessed using 1000 bootstrap replications. Branches corresponding to partitions reproduced in less than 50% bootstrap replicates are collapsed. The percentage of replicate trees in which the associated taxa clustered together in the bootstrap test are shown next to the branches.

To investigate the evolutionary relationships a multiple sequence alignment (MSA) was constructed for the cnidarian TCTPs (Figure 3a). Such alignment resulted in 185 variable residues, 104 of which were parsimony informative over 207 amino acidic residues. Considering the alignment of TCTPs from the sea anemones (*A. viridis*, *E. pallida* and *N. vectensis*) and the stony corals (*A. millepora* and *A. digitifera*) 158 residues were variable, 69 of which were informative. The alignment of TCTPs from sea anemones and hydroid *Hydra* retrieved 185 variable amino acids and 33 informative residues. Similarly, the pairwise comparison between stony corals and *Hydra* showed 162 variable sites and 20 informative residues.

Therefore, AvTCTP showed the highest sequence similarity with the cnidarian homologue from sea anemone *E. pallida* (51.4% identity); while, the identity shared with the homologues from *Hydra* and the sea anemone *N. vectensis* ranged between 20.9% and 24.6%. Higher similarity was shared with TCTPs from the stony coral *A. digitifera* and *A. millepora* (30.2%). Similarly, TCTP from *N. vectensis* shared analogous identities with homologues from stony corals (32.1%), while lower identities (24% and 18%, respectively) were shown with TCTPs from *Hydra.* Finally, MSA for TCTPs from the stony corals *A. digitifera* and *A. millepora* with Hydra, retrieved an identity comprised of between 31% and 22%.

The inferred ML tree showed a topology with branching according to taxonomic differences among *H. vulgaris*, *E. pallida*, *A. viridis*, *N. vectensis*, *A. millepora* and *A. digitifera.* Cluster analysis indicates the presence of four defined groups as suggested by the significant bootstrap values which support the nodes. Branching corresponding to species distinctions (Figure 3b) were retrieved. Thus, the TCTPs from stony corals are primitive with respect to the more complex Hydrozoa class; while the TCTP from sea anemones separates the basal from the derived proteins. 

**Figure 4:**
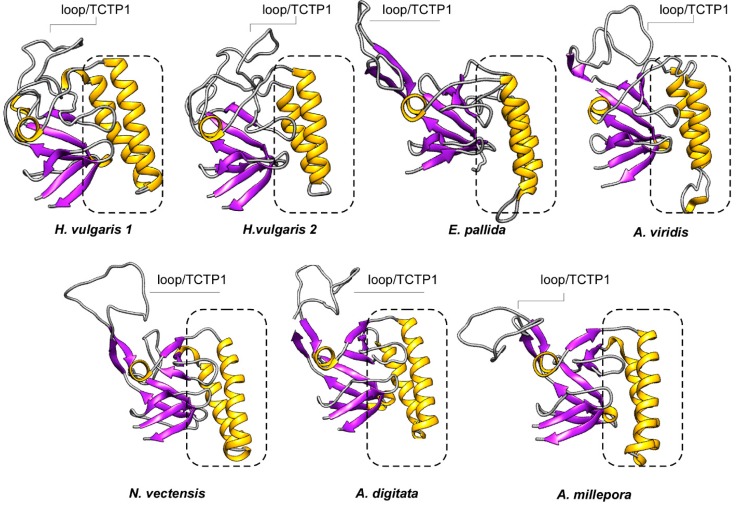
Ribbon diagrams of the cnidarian TCTP structures herein defined. General overview of the 7 TCTPs generated by homology modelling. The 3D structures were created via the Phyre 2 software [41] and rendered by using Chimera package [45]. The proteins were coloured according to secondary structure with beta-sheets in purple and helices in yellow. The dotted rectangles identify the helical hairpin forming the microtubule and Ca^2+^ binding domain.

In order to probe the evolution of protein families, computational approaches were herein used to integrate structural concern with phylogenetic analyses. Thus, we computed the secondary structure elements (SSEs) and the 3D structure of these TCTPs was predicted by homology modelling (Figure 4). The N-term of cnidarian TCTPs showed a high degree of β-strand configurations since usually six β-strands precede three α helices located in the central portion of the proteins. Finally, the C-term resulted structured again in four β-strands. The distribution and number of SSEs in cnidarian homologues resulted similar to those described in previous studies. Interestingly, TCTPs from stony corals and *A. viridis* present a 3_10_ helix located in the Ca^2+^ and microtubule docking site; thus resembling the organization found in human homologue [52].

Analyses of 3D structures showed that cnidarian TCTPs grossly consist of a β-stranded ‘core’ domain usually comprising four beta-sheets and three α helices. H2 and H3 α helices form an alpha-helical hairpin and allocate both the tubulin-binding region and the Ca^2+^-binding motif [53]. A flexible loop located between the core and the helical domain represents a key feature for TCTP proteins, thus revealing a canonical TCTP1 signature. 

An effort was also made to define the accessibility to solvent of the predicted structures. Toward this end, prediction of SSEs was combined with evaluation of RSA of the corresponding models. Based on RSA values, TCTPs from cnidarians were found to mainly consist of exposed residues. We computed an average accessibility of more than 77% per element with RSA between 0.1 and 1. Conversely, buried residues (RSA < 0.1) were retrieved as underrepresented. Thus, this confirm the soluble and hydrophilic nature of TCTPs. 

It is known that exposure to the solvent is negatively correlated with conservation [54,55,56,57,58]. Therefore, these solvent-exposed sites may likely provide tolerances to amino acid substitutions, which in turn allowed variability to accumulate without functional alteration in protein structures. To probe such assumption, conservation analysis among members from the TCTP family was also carried out and TCTPs from different organisms with experimentally determined 3D structures were collected and aligned with cnidarian homologues (see alignments collected in Supplementary Materials).

**Figure 5:**
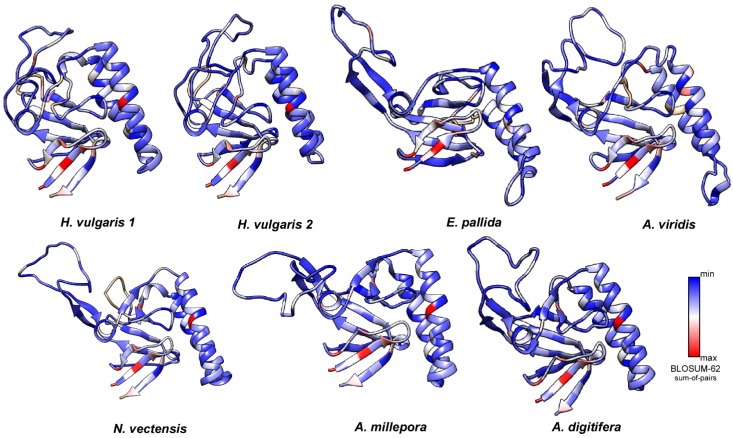
Molecular evolution of cnidarian TCTPs. Three-dimensional models in ribbon representation of various cnidarian homologues with their structure coloured according to the evolutionary conservation of amino acids. The 3D structures were created via the Phyre 2 software and rendered by using Chimera package. Variable positions are presented in blue; while conserved amino acids are showed in red as defined in the color-coding bar.

The cnidarian proteins displayed SSEs mainly consisting in variable residues (Figure 5). Despite the low degree of amino acid conservation compared with highest metazoans, the tertiary structure with the helical core and β sheet core are maintained. Thus, amino acid changes occurred in a way that restraints associated to structural element were accomplished. This was not unexpected, as it occurred for several class of protein including insulins [59], aspartic proteinases [60] and recently it has been described also for members of tissue inhibitor of metalloproteinase (TIMP) family in cnidarians [23].

### 3.3. Phylogenetic Analysis with other Eumetazoa and Structural Inferences

A BLASTp analysis indicated that AvTCTP shows moderate to low identity with other eumetazoan homologues. Thus, to establish homology relationships, sequence similarity analyses were performed at the protein level considering TCTPs from Eumetazoa as listed in Table 1. The homologues from diverse organisms evocative of the Animalia evolution were collected to infer phylogenetic relationship.

The amino acid p-alignment showed a high degree of variability mainly located at the region corresponding to loops between SSEs; while, a major degree of conservation was observed at N- and C-terminal domains.

**Figure 6:**
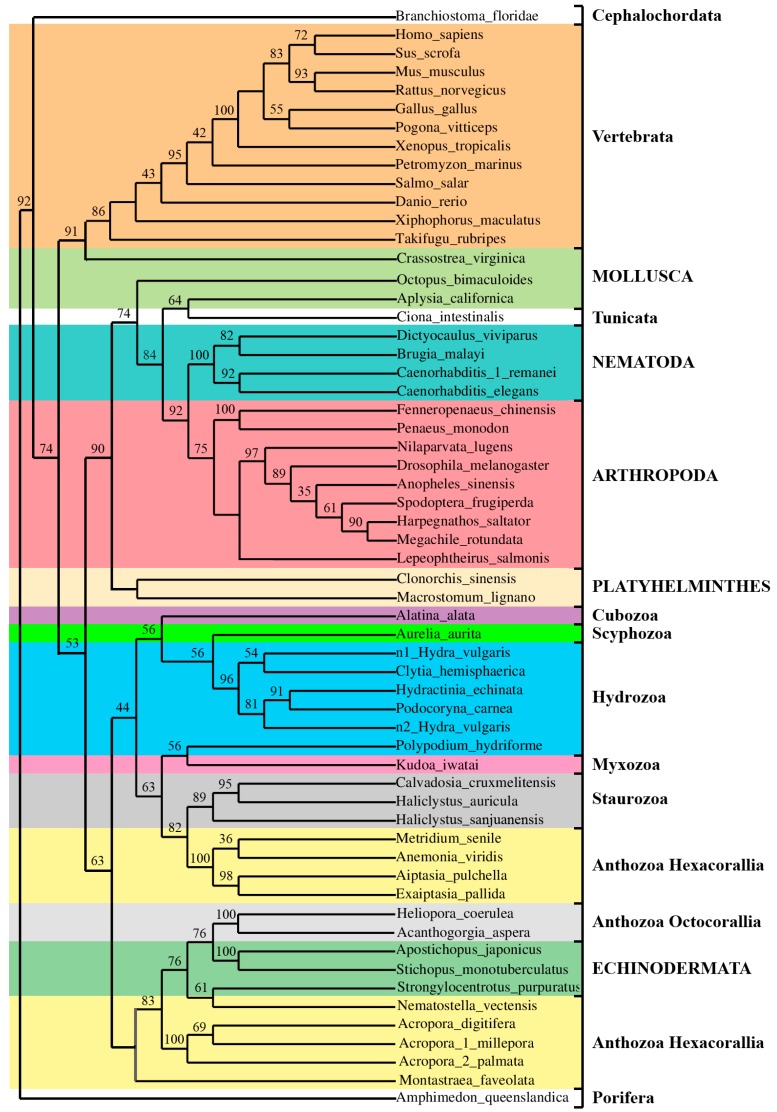
Maximum Likelihood (ML) bootstrap consensus tree based on AvTCTP and homologues from other Eumetazoa. The tree was generated using MEGA 7.0 [39] including TCTP from different species. All the sequences used were obtained from GenBank at NCBI. The bootstrap consensus tree inferred from 1000 replicates is taken to represent the evolutionary history of the taxa analysed. Bootstrap values less than 30% are not shown. The percentage of replicate trees in which the associated taxa clustered together in the bootstrap test (1000 replicates) are shown next to the branches.

Phylogenetic trees were constructed and the generated ML, neighbour joining (NJ) and maximum parsimony (MP) trees resulted were analysed. In Figure 6, the ML tree is shown and the low support values (below 30%) in some nodes suggest the existence of alternative branching arrangements. 

The AvTCTP from sea anemone *A. viridis* was grouped with the homologue from cnidarians, having the shortest evolutionary distances from *Metridium senile*, *Aiptasia pulchella* and *E. pallida* TCTP; accordingly, to taxonomy, all of them belong to Actinaria (sea anemones). Even though the inter-relationships of the classic Cnidaria phylum appear to be resolved, the intra-relationships within subphyla have been troublesome and not well defined. Surprisingly, cluster and topological analysis indicates that the cnidarian clade also contains TCTP homologues from echinoderms belonging to Deuterostoma. Such clustering, including organisms with different evolutionary rates, resulted similar to that constructed for the TIMP protein family showing high degree of variability [23]. Moreover, Hexacorallia including *Acropora* species and *N. vectensis* are more related to Octacorallia with respect to other Hexacorallia; meanwhile *A. viridis* group appeared paraphyletic with Staurozoa. Additionally, the Hydrozoa is more closely related to Scyphozoa and Cubozoa than to Anthozoa. Thus, the transcriptomic survey herein discussed once again confirm the complexity of the Cnidaria phylum that need to be further assessed. Over them, a phylogenetic grouping of TCTPs was, according to taxonomic clustering and with a few exceptions, the same for TCTPs from molluscs that were grouped once with Vertebrata and once with Tunicata. However, the atypical clustering of Tunicata and Cephalochordata herein observed was also retrieved when other protein families were considered [61]. A derived group, incorporated within invertebrates, includes homologues from different organisms belonging to the flatworms, arthropods and nematodes. Finally, as expected, the more derived branch consists of the vertebrate group.

### 3.4. Tissue Expression Pattern of AvTCTP

**Figure 7:**
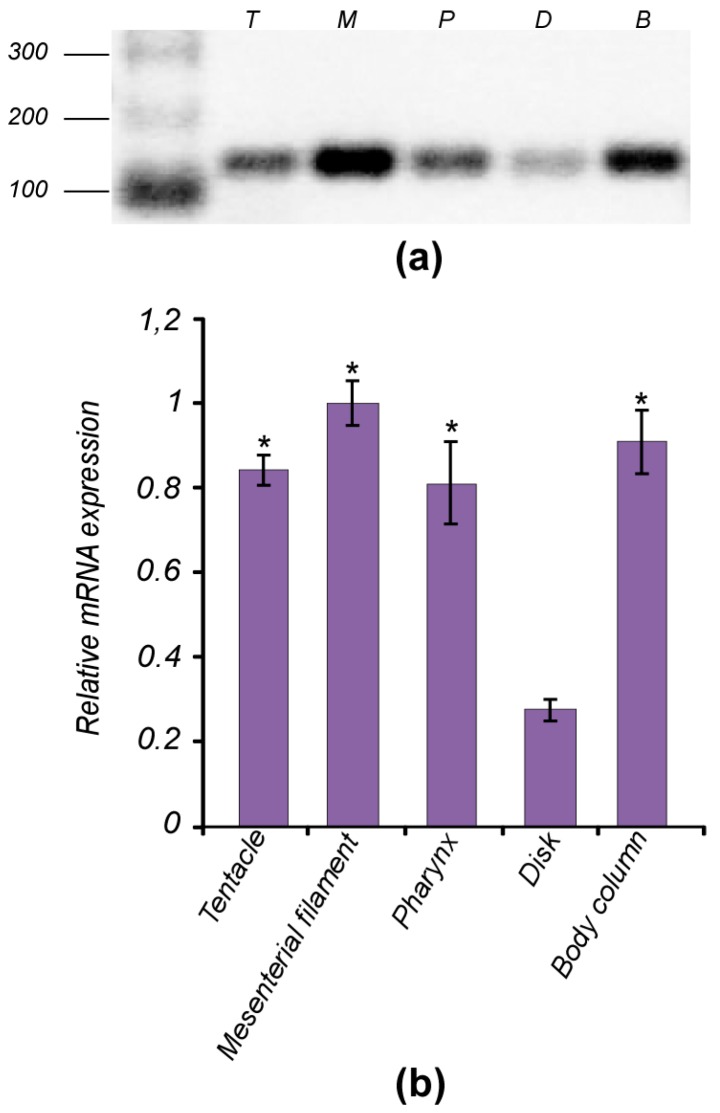
Expression patterns of *AvTCTP* in various tissues. (**a**) Non-quantitative RT-PCR performed to define the tissue specific expression pattern. Total RNA was extracted from different anatomical districts of the sea anemone: tentacles (T), pharynx (P), mesenterial filaments (M) basal disk (D), body column (B). (**b**) RT-qPCR of *AvTCTP* mRNA in the same tissues. Transcript levels in tentacles, pharynx, basal disk and body column were normalised to that of mesenterial filaments. RCC2 and COP-γ were used as housekeeping genes. The results are represented as means ± SD (*n* = 3). Statistical analysis by one-way ANOVA with Tukey’s post-test. * *p* < 0.01.

It has been reported that TCTP homologues, including those from mammals [1], fishes [62,63], molluscs [10,11] and decapods [5,7] are expressed in a variety of cell types and tissues. To evaluate the tissue-specific expression pattern of TCTP also in the basal anthozoan, total RNA was isolated from tentacles, body column, pharynx, mesenterial filaments and basal disk of *A. viridis* and RT-PCR analysis were performed (Figure 7). The *AvTCTP* transcripts were detected in all tissues herein analysed signifying that *AvTCTP* was ubiquitously distributed and constitutively expressed in sea anemone under normal physiological conditions. 

To confirm this expression pattern and evaluate the *AvTCTP* mRNA level in such tissues, RT-qPCR analysis were carried out. Results were congruent with the expression profile previously retrieved; in addition, A*vTCTP* mRNA was detected at highest level in mesenterial filament and section of body column. Moderate level was detected in tentacle and pharynx, while the expression level in the basal disk was relatively low. The Mediterranean Sea anemone presents the diblastic organization of cnidarians consisting of two cell layers—the ectoderm and endoderm—separated by the mesoglea [64,65]. Thus, the mRNA expression pattern of *AvTCTP* is contributed by cells of both layers present in all anatomical districts. This is consistent with a previous work reporting that the TCTP homologue from *Hydra magnipapillata* was expressed in both the ectoderm and the endoderm [15]. The same authors reported the *TCTP* mRNA anatomical distribution by whole-mount in situ hybridization [15]. Interestingly, TCTP transcript was not found in tentacles or basal disk of *H. magnipapillata*; whereas TCTP protein was found at least at the base of the tentacles and the junction between the body column and the foot [15]. Thus, to validate the *AvTCTP* expression in tentacles of the Mediterranean Sea anemone, a BLASTn search was implemented on a tentacle specific RNA library [66]; whereas, no tissue specific transcriptome dataset was available for the basal disk. As a result, at least 100 matching sequences encoding TCTP in this database were retrieved; thus confirming the mRNA expression in the tentacles of this anthozoan. The involvement of TCTP in cell proliferation mechanisms is widely accepted [15]. It is also well known that active proliferation is observed in the body column and in gonads which are situated alongside the mesenterial filaments of cnidarians [67]. Thus, it is possible to suppose that AvTCTP may exert a similar role in sea anemone.

### 3.5. Expression Profiles in Response to Thermal, Chemical and Biotic Challenges

In order to evaluate the TCTP involvement in the stress response, the mRNA levels of *AvTCTP* were profiled after challenges with different abiotic/biotic stresses. Tentacles were dissected and considered as target tissue because of the ability to regenerate without damage on sea anemones viability after dissection.

**Figure 8:**
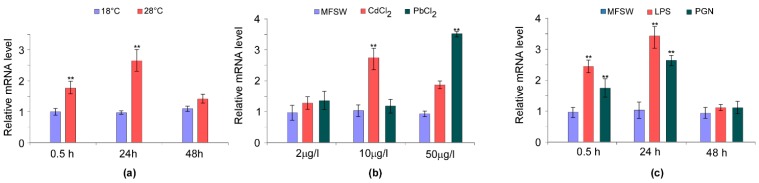
Expression profiles of *AvTCTP* in response to physical and biotic challenges. (**a**) Sea anemones were challenged by hyperthermic conditions at 28 °C, while animals maintained at 18 °C were used as controls. The tentacles were sampled at different time points post-challenge (0.5 h, 24 h and 48 h). (**b**) Sea anemones were exposed to Pb and Cd at different concentrations for 12 h, while animals maintained in MFSW were used as controls. (**c**) Sea anemones were stimulated with different immunogens, while HBSS injected animals were used as controls. Portions of basal disk were sampled from different animals at different time post-challenge (0.5 h, 24 h and 48 h). The expression level of *AvTCTP* was analysed by qRT-PCR with RCC2 and COP-γ as references. The results are represented as means ± standard deviation (SD) (*n* = 3). Statistical analysis by one-way ANOVA with Tukey’s post-test. ** correspond to *p* < 0.005.

It has been reported that sea anemone exposure to increased temperatures results in defects in mesogleal integrity [68] and an inflammatory response. In our study, the exposure of sea anemones to warming (28 °C) boosted the *AvTCTP* mRNA level (Figure 8). In response to heat shock challenge, *AvTCTP* expression was elevated to approximately 1.8-fold greater than the control level within 30 min. The *AvTCTP* transcripts accumulated to a maximum level at 24 h (2.7-fold) and then declined at 48 h. Even after 48 h, the expression was still higher than that of the control (1.4-fold)*.* It has been shown that elevated temperatures affect the redox status in *A. viridis* as hyperthermic stress induced oxidative stress in the sea anemone [16] and photosynthesis dysfunction in the symbiont, thus increasing reactive oxygen species (ROS) formation [17,69]. Additionally, in *A. viridis* the overexpression of small heat shock proteins (HSP) occurs after exposure to thermal stresses [33]. Hence, TCTP might serve as antioxidant and could neutralize increase in ROS levels [1,70]. Thus, it is reasonable to suppose that AvTCTP induction in response to thermal stress may likely occur to cope the ROS production in sea anemone. Because heavy metal ions are known to alter redox milieu—thus making maintenance or reestablishment homeostasis difficult—cadmium (Cd) and lead (Pb) exposures were herein carried out to analyse the mRNA levels of *AvTCTP* after metal challenges (Figure 7b). The AvTCTP mRNA expression increased following the exposure to increasing metal concentration. The transcript was up-regulated after exposure to 2, 10 and 50 µg/L PbCl_2_ and the highest expression (2.8-fold higher than that of the controls) was detected at 10 µg/L. Also, the TCTP expression was induced in response to CdCl_2_ exposure and was over-expressed up to 3.6-fold at 50 µg/L. It has been reported that TCTP transcript levels are affected by calcium ions since a depletion of stored Ca^2+^ results in TCTP mRNA upregulation [71]. Interestingly, Cd exerts a broad range of adverse actions on Ca^2+^-dependent pathways thus altering the calcium-related mechanisms [72]. Additionally, several transcription factor binding sites have been identified within the human TCTP gene promoter, these include the activator protein 1 (AP-1) and cAMP response element-binding (CREB) [73]. It is noteworthy that acute cadmium exposure enhances AP-1 DNA binding and AP-1 protein increased in nucleus under acute heat stress [74] resulting in the overexpression of genes under its control [75]. Moreover, CREB is activated by phosphorylation after acute cadmium exposure [76]. Therefore, it is possible to infer that *AvTCTP* mRNA acute response may likely reflect the AP1 and/or CREB activity under Cd and hyper-thermic acute stress. Although the TCTP members are involved in various stress conditions, no study currently reports the effects of heavy metals on *AvTCTP* mRNA expression in sea anemones. However, our results are consistent with findings reported for coelomocytes from *Eisenia fetida* [77] in which TCTP was found upregulated after suppression subtractive hybridization (SSH) from worms exposed to metallic pollution and for the earthworm *Lumbricus rubellus*, in which *TCTP* expression levels increased significantly after heavy metals exposure [78]. Thus, the mRNA expression profile above described may likely protect sea anemone from the heavy metal exposure.

In the marine environments, sea anemones are continuously exposed also to microbial pathogens. Cnidarians lack an acquired immune system and the innate immunity is activated by pathogen associated molecular pattern (PAMP) including LPS and PGN [79]. This prompted us to profile the AvTCTP mRNA expression in sea anemones exposed to LPS and PGN which were used as Gram-negative and Gram-positive bacterial mimic agents. Sea anemones were separately injected with defined immunogens whereas sea anemones injected with HBSS were used as a control. In our study. Both PAMP exposures resulted in the upregulation of *AvTCTP* mRNA levels. In response to LPS, *AvTCTP* expression was rapidly elevated to approximately 2.5-fold greater than the control level within 6 hours. The transcripts accumulated to a maximum level at 24 h (3.5-fold) and then returned similar to control at 48 h. Similar results were also obtained in PGN injected sea anemones as a quick over expression of *AvTCTP* (up to 1.8-fold) was measured within 6 h. The *AvTCTP* expression increased to a maximum level at 24 h (2.7 fold) and then returned similar to control at 48 h. The outer membrane of Gram-negative bacteria contains LPS as major constituent which ensures structural integrity to the cell and stabilizes the membrane structure [80]. It also represents the principal pathogenic determinant acting as endotoxin and activating the Toll and Toll-like pathways. An abundant coat of PGN constitute the gram-positive cell wall representing 90% of the dry weight of these bacteria [81] and, as occurred for LPS, cell wall components are recognized by host pattern recognition receptors to activate the innate immune system usually triggering the immune deficiency (IMD) pathways [82].

The obtained results show that LPS and PGN as major surface bacterial components are able to upregulate *AvTCTP* mRNA levels; thus suggesting that this factor is significantly involved in the defence mechanisms against Gram-negative and Gram-positive challenges. Analogous results were obtained in haemocytes from shrimps *L. vannamei* and *P. indicus* [8,9] after WSSV challenges and in mollusc *Venerupis philippinarum* [12] in response to *Vibrio anguillarum* stimulation. Thus, is possible to hypothesise that AvTCTP may play a role in the early response to either Gram-negative and Gram-positive pathogens.

## 4. Conclusions

The evolution of TCTP genes, appears to be dynamically represented by a set of complex processes associated with mechanisms of gain and loss of introns and amino acid residues changes. However, the maintenance of the protein 3D structures emerged. Structure maintenance during the evolution is coherent with the roles exerted by TCTPs spanning from apoptosis, microtubule organization, or ion homeostasis. Moreover, data herein presented support the idea that comparative analyses should contemplate both phylogenic and structural considerations. Intriguingly, the repertoire of the cnidarians TCTPs includes genes with complex organisation (several introns interrupting the CDS) as occurred in *A. digitifera* and *H. vulgaris and* intronless genes such as in sea anemones. This multiplicity of gene structures confirms, once again, the molecular complexity of such Phylum.

Additionally, the transcriptional expression pattern retrieved in *A. viridis* suggests a TCTP involvement in the sea anemone defensome. It acts as a ubiquitous factor under physiological condition and related mRNA is modulated during immune and environmental challenges. Thus, it is reasonable to hypothesize its employment as a potential biomarker.

## Figures and Tables

**Table 1:** Specific primers used in this study.

Primers	Sequences (5′–3′)	Amplicon Size (bp)
3′ RACE	GACCGCTTTAATGGAGTCTTC ^F^	-
Fl-TCTP	GAGGTCGAGCTGTACCAACAA ^F^	877
	TTGTTCACGACGAACTTCCTAC ^R^	
RCC2	GGTTCCAAATCCTCCACAAACC ^F^	83
	TGTCCCAATCCGCACGTTAC ^R^	
COP γ	GCCTGTTGGACACCGATGAT ^F^	142
	TGCAAGGCTCTCTCCAGTCC ^R^	
qTCTP	GGATGAAATGTTCGGAGGAAAC ^F^	133
	CATAGTGGGCTGTTCGCTGTAT ^R^	
^F^ Forward primer		
^R^ Reverse primer		

RACE: rapid amplification of cDNA ends; RCC2: Regulator of Chromosome Condensation protein 2; COP-γ: Coatomer subunit gamma; bp: base pair.

**Table 2:** Translationally Controlled Tumour Proteins (TCTPs) from Eumetazoans used for phylogenetic Analysis.

Species	Accession Number	Taxonomic Group
*Homo sapiens*	AAQ01550.1	Mammalia
*Mus musculus*	NP033455.1	Mammalia
*Sus scrofa*	AAL68965.1	Mammalia
*Rattus norvegicus*	NP446319.1	Mammalia
*Gallus gallus*	NP990729.1	Aves
*Xenopus tropicalis*	NP_001008074.1	Amphibia
*Pogona vitticeps*	XP_020652710.1	Reptilia
*Danio rerio*	NP937783.1	Osteichtyes
*Salmo salar*	ACI68686.1	Osteichtyes
*Xiphophorus maculatus*	XP005807723.1	Osteichtyes
*Takifugu rubripes*	XP003962088.1	Osteichtyes
*Petromyzon marinus*	EB084009.1	Agnata
*Branchiostoma floridae*	XP_002592847.1	Cephalochordata
*Ciona intestinalis*	FK151528.1	Tunicata
*Apostichopus japonicus*	ABC87996.1	Echinodermata
*Stichopus monotuberculatus*	AID69538.1	Echinodermata
*Strongylocentrotus purpuratus*	XP795619.2	Echinodermata
*Nilaparvata lugens*	XP_022184371.1	Insecta
*Drosophila melanogaster*	NP001303431.1	Insecta
*Anopheles sinensis*	KFB46001.1	Insecta
*Spodoptera frugiperda*	ADK56158.1	Insecta
*Harpegnathos saltator*	XP011150938.1	Insecta
*Megachile rotundata*	XP_003700051.1	Insecta
*Fenneropenaeus chinensis*	ABB05535.1	Crustacea
*Penaeus monodon*	ACD13588.1	Crustacea
*Lepeophtheirus salmonis*	ACO12977.1	Crustacea
*Octopus bimaculoides*	XP_014780570.1	Mollusca
*Aplysia californica*	XP_005092645.1	Mollusca
*Crassostrea virginica*	XP_022338092.1	Mollusca
*Caenorhabditis elegans*	Q93573.1	Nematoda
*Caenorhabditis remanei*	EFP12520.1	Nematoda
*Dictyocaulus viviparus*	KJH46926.1	Nematoda
*Brugia malayi*	XP001897741	Nematoda
*Clonorchis sinensis*	AAX84199.1	Platyhelmintes
*Macrostomum lignano*	PAA86325.1	Platyhelmintes
*Hydra vulgaris 1*	NM_001309745.1	Hydrozoa
*Hydra vulgaris 2*	XM_002157314.3	Hydrozoa
*Hydractinia echinata*	DT623491.1	Hydrozoa
*Podocoryna carnea*	DY451741.1	Hydrozoa
*Clytia hemisphaerica*	FP985759.1	Hydrozoa
*Polypodium hydriforme*	GBGH01019625.1	Hydrozoa
*Kudoa iwatai*	GBGI01001069.1	Myxozoa
*Aurelia aurita*	GBRG01251580.1	Scyphozoa
*Alatina alata*	GEUJ01004399.1	Cubozoa
*Haliclystus sanjuanensis*	HAHB01030183.1	Staurozoa
*Haliclystus auricula*	HAHA01057349.1	Staurozoa
*Calvadosia cruxmelitensis*	HAHC01090444.1	Staurozoa
*Acanthogiorgia aspera*	GETB01037007.1	Anthozoa Octocorallia
*Heliopora coerulea*	IABP01022130.1	Anthozoa Octocorallia
*Acropora digitifera*	XP_015766473.1	Anthozoa Hexacorallia
*Acropora millepora*	JR988252.1	Anthozoa Hexacorallia
*Acropora palmata*	GW212294.1	Anthozoa Hexacorallia
*Montastraea faveolata*	GW258989.1	Anthozoa Hexacorallia
*Nematostella vectensis*	XM_001624752.1	Anthozoa Hexacorallia
*Metridium* *senile*	FC834313.1	Anthozoa Hexacorallia
*Anemonia viridis*	FK734027.1	Anthozoa Hexacorallia
*Aiptasia pulchella*	CK662981.1	Anthozoa Hexacorallia
*Exaiptasia pallida*	XP_020911835.1	Anthozoa Hexacorallia
*Amphimedon qeenslandica*	XP_003382650.1	Porifera

**Table 3:** TCTPs from Cnidarians.

Organism	Protein Length ^a^	Molecular Weight ^b^	pI ^c^	Templates ^d^
*A. millepora*	173	19,436.99	4.60	1H6Q (*S. pombe*)
				2KWB (*C. elegans*)
				1YZ1 (*H. sapiens*)
				1TXJ (*P. knowlesi*)
*A. digitifera*	173	19,392.98	4.65	1H6Q (*S. pombe*)
				2KWB (*C. elegans*)
				1YZ1 (*H. sapiens*)
				1TXJ (*P. knowlesi*)
*A. viridis*	180	20,553.34	4.54	1H6Q (*S. pombe*)
				2KWB (*C. elegans*)
				1YZ1 (*H. sapiens*)
*N. vectensis*	181	20,715.78	4.78	1H6Q (*S. pombe*)
				2KWB (*C. elegans*)
				1YZ1 (*H. sapiens*)
*E. pallida*	185	21,076.87	4.73	1H6Q (*S. pombe*)
				2KWB (*C. elegans*)
				1YZ1 (*H. sapiens*)
*H. vulgaris 1*	184	20,950.68	4.76	1H6Q (*S. pombe*)
				2KWB (*C. elegans*)
				1YZ1 (*H. sapiens*)
				1TXJ (*P. knowlesi*)
*H. vulgaris 2*	180	20,854.76	5.48	1H6Q (*S. pombe*)
				2KWB (*C. elegans*)
				1YZ1 (*H. sapiens*)
				1TXJ (*P. knowlesi*)

^a^ Length (No. of amino acids) of the deduced protein; ^b^ Molecular weight of the deduced polypeptide in Dalton; ^c^ Isoelectric point of the deduced protein; ^d^ PDBs (X-ray or NMR-based structure) used to model cnidarian TCTPs. *Schizosaccharomyces pombe*, *S. pombe; Plasmodium knowlesi*, *P. knowlesi*.

## Supplementary Materials

The following are available online at www.mdpi.com/2073-4425/9/1/30/s1. Alignment (.meg format) of cnidarian TCTPs with experimentally determined 3D structures.
